# Microstructural Origin of Nonmonotonic Piezoresistivity in Polymer Nanocomposites

**DOI:** 10.1002/advs.202504381

**Published:** 2025-06-23

**Authors:** Ting Yui Wong, Kui Lin, Tao Yu, Fangxin Zou

**Affiliations:** ^1^ Department of Civil and Environmental Engineering The Hong Kong Polytechnic University Hung Hom Kowloon Hong Kong SAR 999077 China; ^2^ Department of Aeronautical and Aviation Engineering The Hong Kong Polytechnic University Hung Hom Kowloon Hong Kong SAR 999077 China

**Keywords:** polymer nanocomposite, piezoresistivity, strain sensing, resistance‐strain inversion, barrier‐crossing mechanism

## Abstract

Incorporating conductive nanomaterials into polymers yields a new class of piezoresistive strain‐sensing materials. While possessing monotonic resistance‐strain behavior is a fundamental requirement for any material used for strain sensing, polymer nanocomposites frequently exhibit nonmonotonic resistance responses under strain, which limits their application prospects. In this study, physical experiments and molecular dynamics simulations are performed to determine a feasible solution to overcome this limitation. The corresponding results demonstrate that regulating the initial inter‐nanofiller junction geometry imparts complete control over the monotonic piezoresistive behavior of polymer nanocomposites. Mechanistically, monotonically increasing resistance responses under tension can be achieved by promoting active diffusion that causes van der Waals force‐driven barrier crossing of nanofillers (resulting in direct contact between nanofillers, e.g., at elevated curing temperatures) during curing; thus, during deformation, nanofillers primarily move away from one another. Conversely, suppressing diffusion during curing causes barrier crossing of nanofillers, which results in resistance reduction, under deformation owing to stress‐driven local rearrangement of polymer molecules in heterogeneous shear transformation zones. The mechanistic insights provided by this study can guide the design of next‐generation, advanced strain‐sensing materials in the future.

## Graphical abstract

When polymer nanocomposites are subjected to tensile loading, the electrical resistance‐strain relationship may be nonmonotonic, hampering the use of these materials in real‐world applications. This challenge is addressed through the collaborative use of physical experimentation and computer simulations, establishing the inter‐nanotube junction geometry as the fundamental microstructural parameter that dictates the monotony of the piezoresistive behavior.

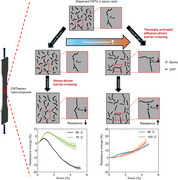

## 1 Introduction

To date, various studies have been conducted to assess the feasibility of incorporating conductive nanomaterials such as carbon nanotubes (CNTs) and graphene as reinforcements in polymer matrices and thus develop advanced multifunctional composite materials.^[^ ^1^, ^2^, ^3^, ^4^ ^]^ Piezoresistivity, a key electromechanical property of conductive nanomaterials, enables strain‐sensing capabilities.^[^ ^5^, ^6^, ^7^, ^8^ ^]^ For strain sensing materials, monotonic piezoresistive behavior is essential; otherwise, ambiguous sensing outcomes would arise, where one resistance measurement could indicate multiple strain values. Nonmonotonic piezoresistive behavior has often been observed in polymer nanocomposites, severely affecting the application prospects of this material class.^[^ ^9^ ^]^

Boland et al. associated the nonmonotonic piezoresistive behavior of a graphene‐filled lightly crosslinked polysilicone nanocomposite with the viscoelasticity of the polymer.^[^ ^10^ ^]^ They proposed that, during mechanical deformation, a low matrix viscosity enables the movement of nanofillers by diffusion or under an applied electric field, leading to nanofiller network relaxation and a decrease in resistance. Similar undesirable piezoresistive behavior has also been observed in other elastomer nanocomposites.^[^ ^11^, ^12^ ^]^ Beyond elastomers, which are generally highly viscoelastic, thermosetting polymers, another type of extensively explored matrix material, have shown large variations in the monotony of their piezoresistive behaviors.^[^ ^13^, ^14^, ^15^, ^16^, ^17^ ^]^ Thus far, the emergence of nonmonotonic piezoresistive behavior has been mainly attributed to matrix plasticity, transverse nanofiller movement under Poisson's effect, and nanofiller network rearrangement under a large strain.^[^ ^9^ ^]^

Considering the broad variation in microstructure across different polymer nanocomposites and the complex interactions between the constituents, controllability over the monotony of the piezoresistive behavior has not been demonstrated. The most extensively studied factor for achieving controllability is the nanofiller content; however, the experimental data are not consistent, and its impact on the monotony is rather limited.^[^ ^10^, ^15^, ^16^, ^17^ ^]^ Meeuw et al.^[^ ^14^ ^]^ investigated the effect of nanofiller alignment. Their results indicated that the alignment of nanofillers along the loading direction by an electric field significantly enhanced the monotony, establishing the importance of network morphology; however, the underlying mechanism was not explored. Predicting and controlling the piezoresistive behavior of polymer nanocomposites remains an important challenge. Numerical simulations have helped shed light on the microstructural origin of hysteretic piezoresistive responses,^[^ ^18^ ^]^ but that of nonmonotonic responses has not been adequately explained. The major obstacles to achieving controllability over the monotony of the piezoresistive behaviors of polymer nanocomposites are the complex microstructures of the constituents and the sophisticated interactions between them, which hamper the effective control of the changes in the nanofiller network morphology in response to mechanical deformation.^[^ ^1^ ^]^

In this study, we employed both physical experiments and coarse‐grained molecular dynamics (CGMD) simulations to address this challenge, demonstrating that complete control over the monotony of the piezoresistivity of polymer nanocomposites can be achieved by regulating the initial inter‐nanofiller junction geometry. Our experimental results, together with numerical evidence, suggest that during curing, barrier crossing of nanofillers due to van der Waals (vdW) interactions occurs, leading to direct contact between nanofillers. In addition, the initial inter‐nanofiller junction geometry of the developed nanofiller network is directly determined by the degree of diffusion. During deformation, stress‐driven local rearrangements of polymer molecules in heterogeneous shear transformation zones can trigger barrier crossing of the nanofillers, further modifying the inter‐nanofiller junction geometry. For a nanofiller network that is developed under active diffusion, the agglomerated nanofillers would, by and large, move away from each other during deformation, causing the overall resistance to increase. However, when diffusion is suppressed, re‐agglomeration of the nanofillers occurs during deformation, resulting in a reduction in resistance. The initial inter‐nanofiller junction geometry, which governs the monotony of the piezoresistive behavior of polymer nanocomposites, can be modulated by the curing temperature, polymer molecular structure, and alignment of the nanofiller, all of which influence nanofiller mobility during curing.

## 2 Results and Discussion

### 2.1 Curing Dependent Piezoresistivity

**Figure 1:**
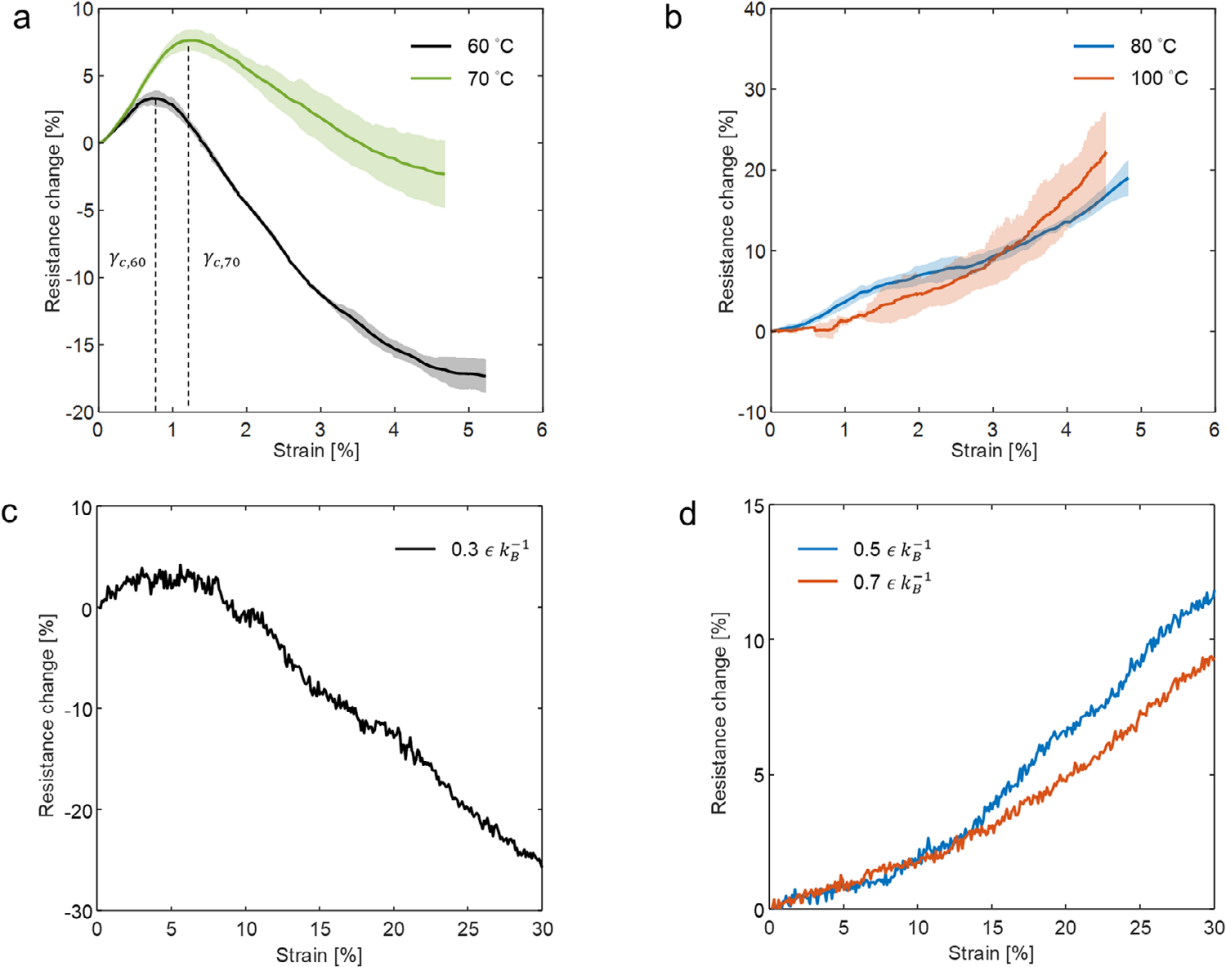
Experimental and CGMD simulation results of piezoresistive behaviors of polymer nanocomposites. a,b) Experimental resistance change versus strain relationships of CNT/epoxy nanocomposites cured at 60, 70, 80, and 100 °C for 6, 4, 3, and 1 h, respectively, followed by applying post‐curing treatment at 125 °C for 3 h. At least three specimens were tested for each sample and the mean response is plotted (shaded area: ±1 standard deviation). c,d) Simulated resistance change versus strain relationships of CNT/epoxy nanocomposites cured at 0.3, 0.5, or 0.7 $\varepsilon k_{B}^{-1}$. The CGMD model parameters are CNT concentration = 5 vol.%, number of beads per CNT = 100, CNT diameter = 1.0 *σ*, CNT waviness in terms of maximum deviation angle = 10°, and simulation cell size = 150 *σ*. The liquid epoxy structure is a two‐bead mixture.

We fabricated CNT/epoxy nanocomposites by curing DER332 epoxy resin, containing well‐dispersed multiwalled CNTs, at 60, 70, 80, and 100 °C for 6, 4, 3, and 1 h, respectively, followed by applying post‐curing treatment at 125 °C for 3 h (Figure S5a and Table S1, Supporting Information). The samples were then loaded under tension until failure (Figure S5b, Supporting Information). The piezoresistive behavior transformed from nonmonotonic to monotonic as the curing temperature increased (**Figure** **1**a,b), while the mechanical properties remained similar (Figure S6, Supporting Information). For the 60 and 70 °C samples, the resistance first increases with strain, and after reaching the maximum at the critical strain (*γ*_c_), decreases until the failure of the specimen, i.e. inversion of piezoresistivity. The *γ*_c_ of the 70 °C sample is larger than that of the 60 °C sample. Upon reaching 80 °C, the resistance monotonically increased until failure. We previously reported a similar observation, where a lower critical temperature for the monotonic transition was observed with another epoxy matrix (Figure S7, Supporting Information).^[^ ^19^ ^]^ The CNT content is fixed at 0.3 wt.%, which is just beyond that in a percolation region in a similar material system (Figure S8, Supporting Information), given that the monotony of the piezoresistive behavior or *γ*_c_ is insensitive to the CNT content (Figure S9, Supporting Information).

The transformation of the piezoresistive behavior with the curing temperature suggests differential changes in the CNT network configuration under tensile deformation (Figure 1c). The electrical properties and piezoresistive behavior of polymer nanocomposites, including CNT/epoxy nanocomposites, are largely determined by the quantum tunneling effect, which depends on the distance between adjacent nanofillers.^[^ ^20^ ^]^ Under tensile deformation, the nanofillers move apart, thereby increasing the inter‐nanofiller distance and electrical resistance. However, the inversion of piezoresistivity indicates processes that reduce the inter‐nanofiller distance, likely due to molecular‐level phenomena at the nanometer scale involving nanofiller‐matrix interactions. The unclear microstructural origin of nonmonotonic piezoresistivity necessitates a mechanistic study to advance our understanding and achieve the controllability of piezoresistivity in polymer nanocomposites.

To accomplish this task, we employed molecular dynamics (MD) simulations and constructed a CGMD model of a periodically repeating unit cell representing CNT/epoxy nanocomposites.^[^ ^18^ ^]^ Details of the CGMD model and simulation methods are provided in Section S1 (Supporting Information). Since our aim is to pinpoint the microstructural origin, we adopt the reduced units formalism, expressing all physical quantities as multiples of fundamental quantities: mass (*m*), energy (*ϵ*), distance (*σ*), and Boltzmann constant (*k*_B_), each set to one, which allow us to focus on the relative interactions and dynamics.^[^ ^21^, ^22^, ^23^ ^]^ The simulated resistance‐strain curves reproduced the experimentally observed temperature dependence of the piezoresistive behavior of the CNT/epoxy nanocomposites (Figure 1d,e). Details of the resistor network model for calculating the resistances of the CNT networks in the CGMD simulations are provided in Section S2 (Supporting Information). The curve is nonmonotonic for 0.3 $\varepsilon k_{B}^{-1}$ and becomes monotonic for 0.5 and 0.7 $\varepsilon k_{B}^{-1}$. The resemblance between the simulations and experiments, although qualitative owing to the simplification of molecular details into beads and springs in this study, suggests that the microstructural origin of the nonmonotonic piezoresistive behavior can be traced through analysis of the simulations. Quantitative alignment with experimental data, which requires further calibration of the model parameters, will be attempted in our future investigations.

### 2.2 Inter‐Nanofiller Junction Geometry and Microstructural Origin

**Figure 2:**
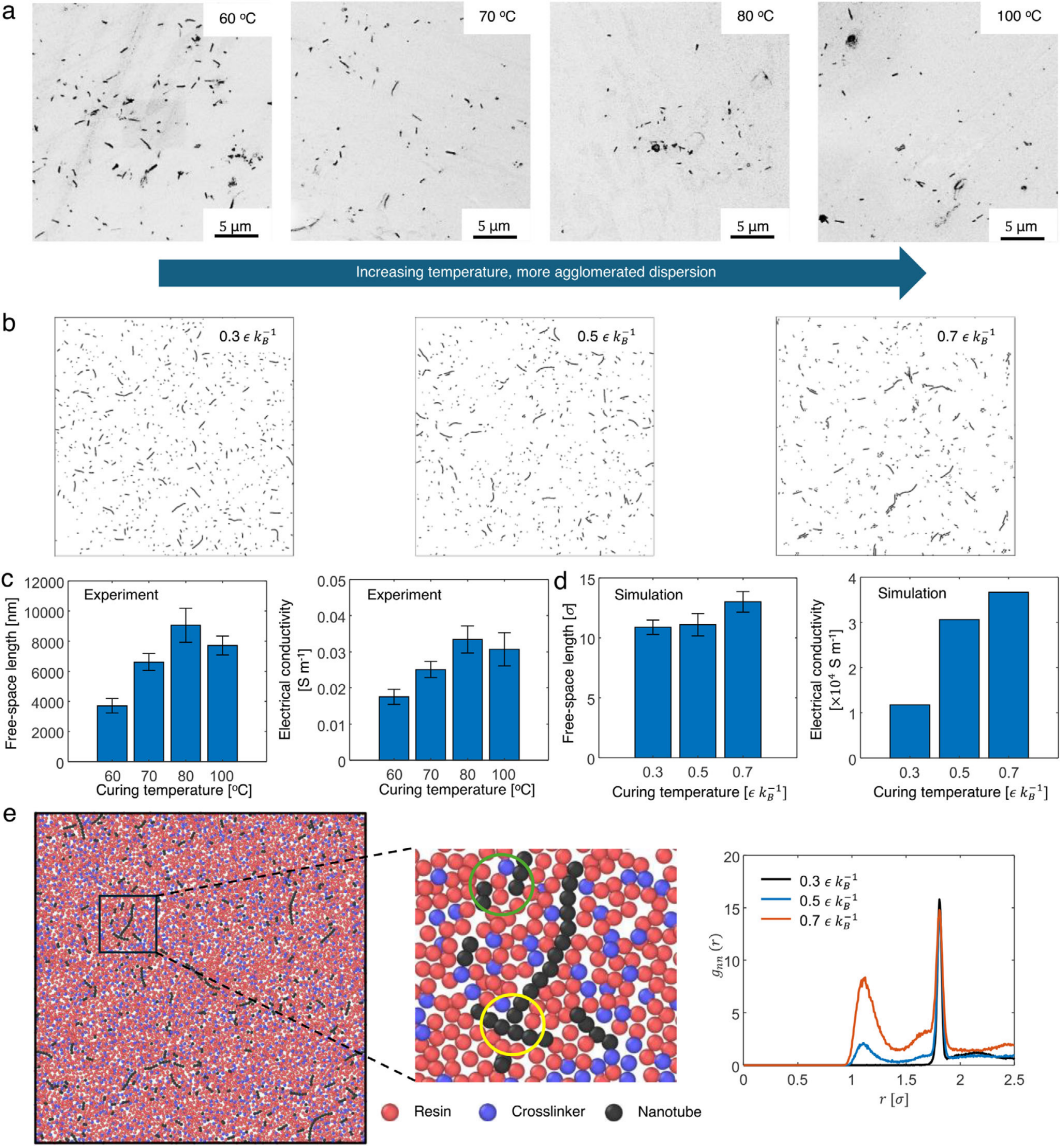
Experimental and CGMD simulation results of inter‐nanofiller junction geometries and electrical conductivities of polymer nanocomposites. a,b) FESEM images and CGMD model slices showing the morphologies of CNT networks developed under different curing temperatures. Black and white are inverted in FESEM images for better visibility. c,d) Experimental and simulation results of the free‐space lengths and electrical conductivities of CNT/epoxy nanocomposites cured at different temperatures. For the experimental results, at least four specimens were tested for each sample. For the simulation results, nine slices from each model were used for free‐space length analysis (error bar: ±1 standard deviation). e) CGMD model slice demonstrating the inter‐nanotube junction morphology in direct contact or with polymer barrier which corresponds to 1*σ* or 2*σ* peak in the RDF of nanotube beads. The CGMD model setup is CNT concentration = 5 vol.%, number of beads per CNT = 200, CNT diameter = 1.0 *σ*, CNT waviness in terms of maximum deviation angle = 10°, and simulation cell size = 100 *σ*. The liquid epoxy structure is two‐bead mixture.

Upon increasing the curing temperature, the nanotube microstructure became more visibly agglomerated, as shown in the field‐emission scanning electron microscopy (FESEM) images (**Figure** **2**a) and slice images of the CGMD simulation cells (Figure 2b). The dispersion state was quantitatively characterized using free‐space length analysis and electrical conductivity measurements.^[^ ^24^ ^]^ More details on dispersion state characterization are provided in Section S3 (Supporting Information). The agglomerated dispersion leads to a larger free‐space length and facilitates the formation of electrical conduction paths, resulting in higher electrical conductivity.^[^ ^25^ ^]^ The free‐space lengths and the electrical conductivities are higher for the 80 and 100 °C or 0.5 and 0.7 $\varepsilon k_{B}^{-1}$ samples which exhibit monotonic piezoresistive behavior (Figure 2c,d). These observations are consistent with the previous experimental results.^[^ ^24^ ^]^ At a low temperature 0.3 $\varepsilon k_{B}^{-1}$, a strong radial distribution function (RDF) peak is detected near 2*σ* for the nanotube beads (*g*_nn_(*r*)) before deformation, representing a perfectly dispersed state with all nanotubes surrounded by the polymer beads and all inter‐nanotube junctions formed with a polymer layer barrier (green circle in Figure 2e). A peak near 1*σ*, which represents inter‐nanotube junctions in direct contact (yellow circle in Figure 2e), emerges at 0.5 $\varepsilon k_{B}^{-1}$. The 1*σ* and 2*σ* peaks compete with each other; as the curing temperature increases, the height of the former increases, whereas that of the latter shrinks, suggesting the occurrence of thermally activated processes that affect the inter‐nanotube junction geometry.^[^ ^10^ ^]^ Notably, the free‐space length and conductivity of the 100 °C sample were lower than those of the 80 °C sample. While this observation seems to counteract our claim that thermally activated barrier‐crossing events drive changes in the inter‐nanotube junction geometry during curing, we provide a possible explanation for the validity of our claim based on the shorter gelation time under more rapid crosslinking.^[^ ^10^, ^26^ ^]^ Further details on the influence of crosslinking dynamics on the dynamic percolation of nanofillers are provided in Section S5 (Supporting Information).

We ascribe the changes in the initial inter‐nanotube junction geometry to nanotube propagation via diffusion in a viscous medium, such as epoxy resin;^[^ ^10^ ^]^ this phenomenon reflects thermally activated energy barrier crossing.^[^ ^27^ ^]^ Because diffusion is suppressed at low temperatures (0.3 $\varepsilon k_{B}^{-1}$), the inter‐nanotube junction geometry remains unchanged unlike the dispersed state (Figure 2e; Figure S1, Supporting Information). Diffusion is thermally activated at higher temperatures (0.5 or 0.7 $\varepsilon k_{B}^{-1}$), allowing barrier‐crossing transitions between 1*σ* and 2*σ* peaks. The vdW interaction between nanotubes plays a vital role in the occurrence and growth of 1*σ* peak, as the attractive force brings the nanotube beads closer from 2*σ* peak to the energy minimum of the Lennard‐Jones potential at 2^1/6^ ≈ 1.12*σ*.^[^ ^25^, ^28^ ^]^ For the electrical properties, electron transport between nanotubes is conducted through the insulating barrier under the tunneling mechanism, which is commonly described by the Simmons formula^[^ ^29^ ^]^ or the Landauer–Büttiker formula.^[^ ^30^ ^]^ A common feature of these formulae for describing the tunneling resistance between nanotubes is the exponential dependence of the inter‐nanotube distance. When thermally activated diffusion induces the agglomeration of nanotubes at higher temperatures, the tunneling resistance is reduced owing to the smaller inter‐nanotube separation. Therefore, even with only a small portion of nanotube beads transitioning from 2*σ* peaks to 1*σ* peak through barrier‐crossing at 0.5 $\varepsilon k_{B}^{-1}$, as indicated by the similar free‐space lengths for 0.3 and 0.5 $\varepsilon k_{B}^{-1}$ (Figure 2d) and the RDF plots (Figure 2e), the emergence of direct contact between nanotube beads results in significantly higher electrical conductivity.

**Figure 3:**
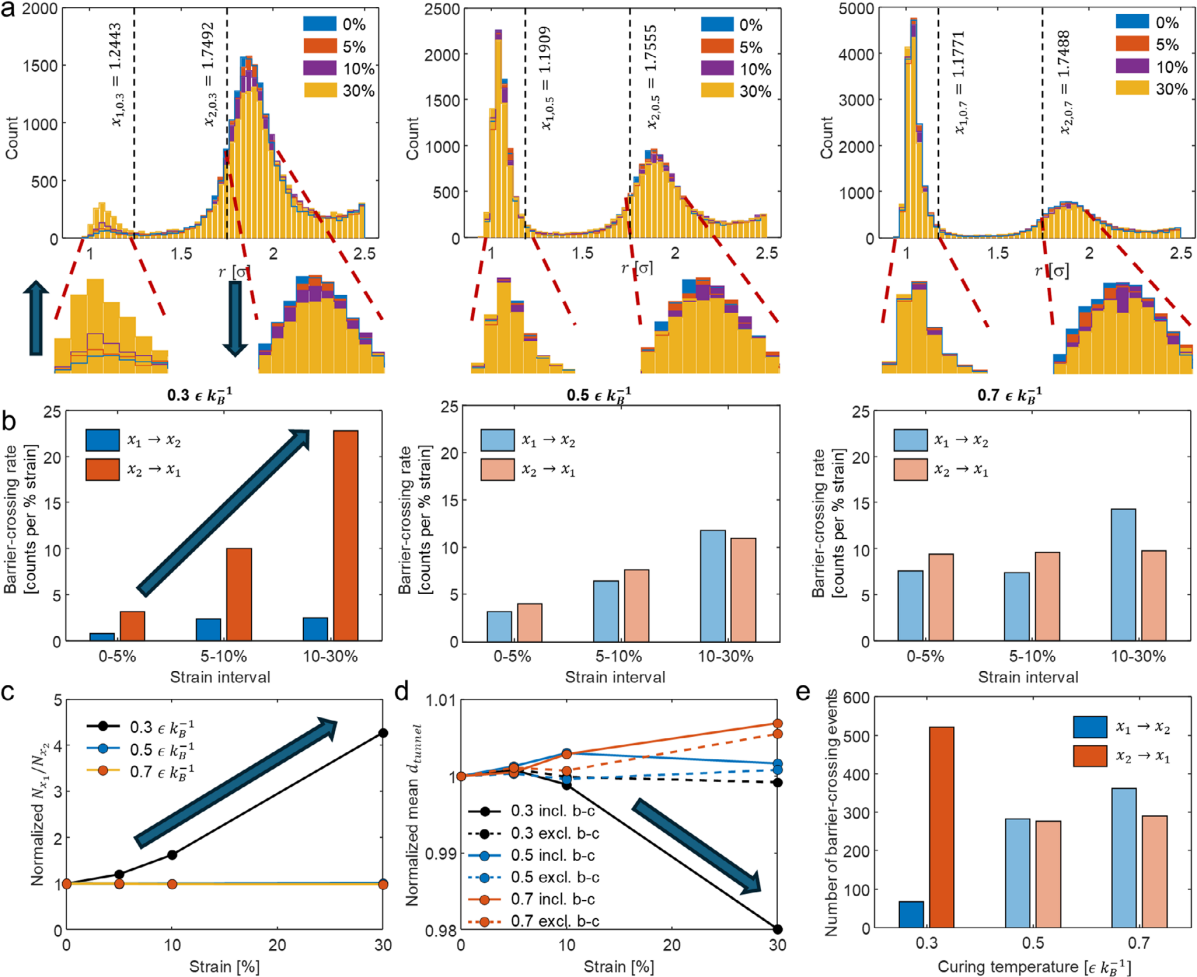
CGMD simulation results and analysis of changes in inter‐nanofiller junction geometry under tensile deformation (key results related to the nonmonotonic piezoresistive behavior are indicated by darker colors or arrows). a) Evolutions of inter‐nanotube tunneling distance distribution with respect to strain. Positions of peaks are determined by Gaussian fitting. b) Rates of barrier‐crossing events from *x*_1_ to *x*_2_ and vice versa. c) Ratios between numbers of inter‐nanotube junctions with distance smaller than *x*_1_ and with distance greater than *x*_2_, normalized with respect to the initial ratio at 0% strain. d) Changes in normalized mean *d*_tunnel_ including or excluding junctions exhibiting barrier‐crossing during stretching. e) Total numbers of barrier‐crossing events. The curing temperatures are 0.3, 0.5, and 0.7 $\varepsilon k_{B}^{-1}$. The CGMD model setup is CNT concentration = 5 vol.%, number of beads per CNT = 100, CNT diameter = 1.0 *σ*, CNT waviness in terms of maximum deviation angle = 10°, and simulation cell size = 150 *σ*. The liquid epoxy structure is two‐bead mixture.

The piezoresistivity of polymer nanocomposites is primarily influenced by alterations in filler network configuration under mechanical deformation, governed by the distance between neighboring nanofillers.^[^ ^9^ ^]^ Under tensile deformation, nanofillers within the nanocomposite move apart. This separation widens, increasing the tunneling resistance and overall electrical resistance. To determine the microstructural origin of nonmonotonic piezoresistive behavior, we monitored changes in the tunneling distance (*d*_tunnel_) distribution, reflecting the movement of junction‐forming nanotube pairs in CGMD simulations. The bistable feature of distance distribution was preserved, suggesting inter‐nanotube geometry was governed by vdW interactions during deformation. We observe that in the 0.3 $\varepsilon k_{B}^{-1}$ case, considerable amount of inter‐nanotube junctions originally with polymer layer barrier (at 2*σ* peak) become in direct contact (at 1*σ* peak) through barrier‐crossing process (**Figure** **3**a), leading to reduced inter‐nanotube distance and decrease in resistance. In particular, the increase and decrease in the numbers of counts under the 1*σ* and 2*σ* peaks, respectively, coincide with the inversion of piezoresistivity, which occurs within the strain range of 5–30% (Figure 1d). Conversely, for the 0.5 and 0.7 $\varepsilon k_{B}^{-1}$ cases that exhibit monotonic piezoresistive behavior, the number of counts under the 1*σ* peak does not increase.

Barrier‐crossing events induce tunneling distance transitions between 1*σ* and 2*σ* peaks. We define the middle two‐thirds of the distance between the two peaks as the barrier region^[^ ^27^ ^]^; therefore, the two boundaries for the two peaks are *x*_1_ and *x*_2_. Identifying changes in inter‐nanotube junction distance from smaller than *x*_1_ to greater than *x*_2_ (*x*_2_ → *x*_1_) and vice versa (*x*_1_ → *x*_2_) as transition paths, we calculate the rate of barrier‐crossing events with respect to strain at discrete strain intervals, which could be correlated to the observed changes in the tunneling distance distribution. The increase and decrease in the numbers of counts under the 1*σ* and 2*σ* peaks, respectively, in the 0.3 $\varepsilon k_{B}^{-1}$ case, which cause the inversion of piezoresistivity, can be attributed to the increase in the *x*_2_ → *x*_1_ crossing rate, whereas the *x*_1_ → *x*_2_ crossing rate remains unchanged (Figure 3b). The former increases from approximately three times that of the latter to approximately ten times. The imbalance between the two types of barrier‐crossing events increases the ratio between the number of junctions in the 1*σ* peak and that in the 2*σ* peak ($N_{x_{1}}/N_{x_{2}}$); this ratio is normalized with respect to the initial ratio at 0% strain (Figure 3c). When the two crossing rates hold in fair balance in the 0.5 and 0.7 $\varepsilon k_{B}^{-1}$ cases, the ratio $N_{x_{1}}/N_{x_{2}}$ remains similar during deformation. Even though the *x*_2_ → *x*_1_ crossing rate is mostly higher, the mean *d*_tunnel_ shows an increasing trend in the 0.5 and 0.7 $\varepsilon k_{B}^{-1}$ cases, as well as at the initial stage in the 0.3 $\varepsilon k_{B}^{-1}$ case (Figure 3d). This result indicates that the nanotubes generally moved apart, leading to positive piezoresistivity. When the junctions exhibiting barrier crossing are excluded, the mean *d*_tunnel_ does not decrease in the 5–30% strain range in the 0.3 $\varepsilon k_{B}^{-1}$ case. The effect of the imbalance between the two types of barrier‐crossing events on the mean *d*_tunnel_ is apparent. The *x*_2_ → *x*_1_ crossing is the major contributor to the decrease in *d*_tunnel_ and hence the nonmonotonic piezoresistive behavior. For the monotonic 0.5 and 0.7 $\varepsilon k_{B}^{-1}$ cases, the influence of barrier‐crossing events on the change in *d*_tunnel_ is much smaller, since the rates of the two opposite events are similar. The total number of barrier‐crossing events shows a distinct correlation with $N_{x_{1}}$ and $N_{x_{2}}$, i.e., more junctions in the 1*σ* (or 2*σ*) peak results in more *x*_1_ → *x*_2_ (or *x*_2_ → *x*_1_) barrier‐crossing events (Figure 3e), whereas the initial $N_{x_{1}}$ and $N_{x_{2}}$ are influenced by thermally activated diffusion as elucidated earlier.

**Figure 4:**
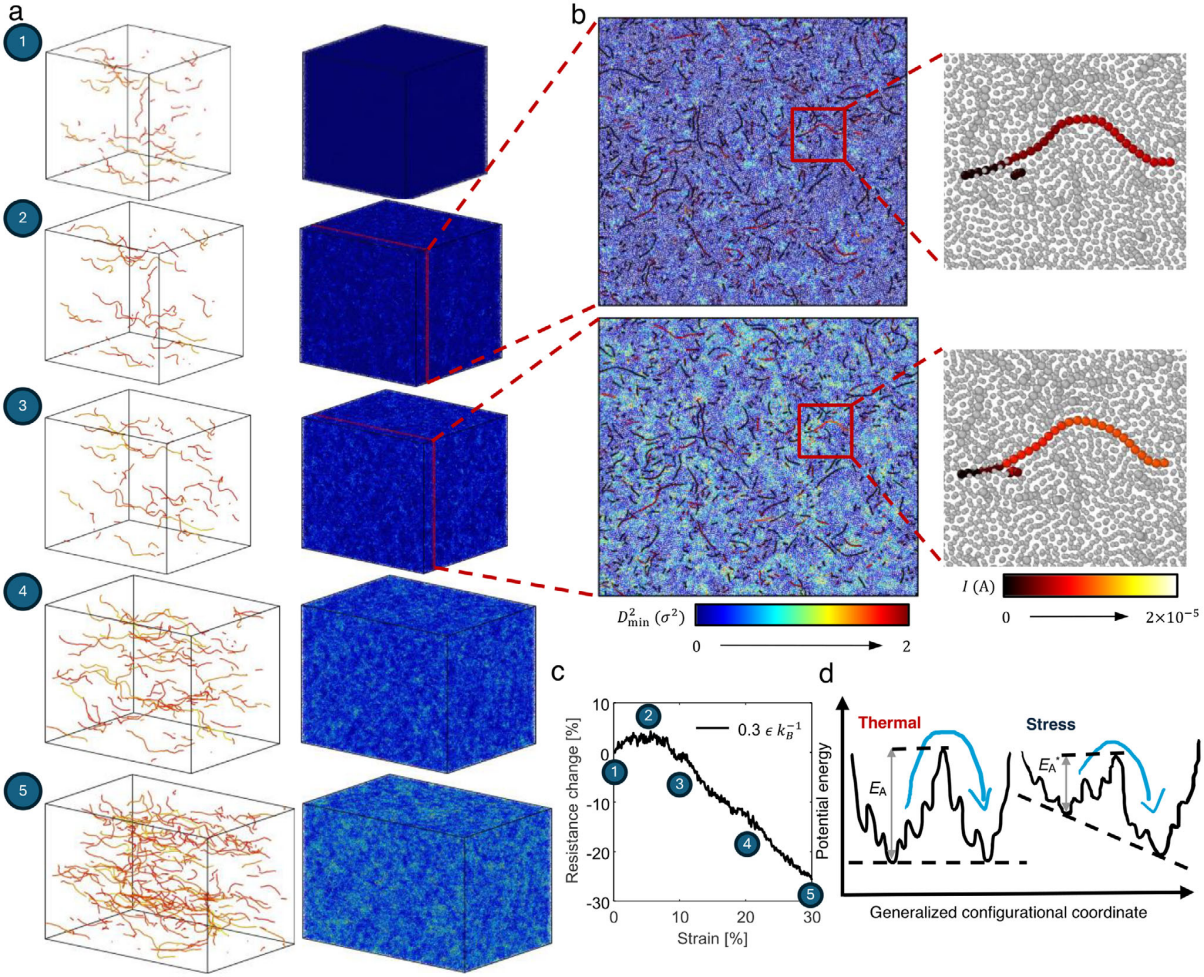
Correlation between local molecular rearrangement and barrier‐crossing process that trigger nonmonotonic piezoresistive behavior under tensile deformation. a) Evolution of the main current‐carrying portion of the CNT network and spatial distribution of nonaffine displacement ($D_{\min}^{2}$) with respect to strain. b) Closeups illustrate the association between local barrier‐crossing events and inversion of resistance change. c) Simulated resistance change versus strain relationships of CNT/epoxy nanocomposite cured at 0.3 $\varepsilon k_{B}^{-1}$. d) Schematic of a fractal PEL and barrier‐crossing processes of a STZ triggered by thermal and stress activations. The CGMD model setup is CNT concentration = 5 vol.%, number of beads per CNT = 100, CNT diameter = 1.0 *σ*, CNT waviness in terms of maximum deviation angle = 10°, and simulation cell size = 150 *σ*. The liquid epoxy structure is two‐bead mixture.

Understanding the distinction between barrier‐crossing events that occur prior to and during deformation is crucial. As discussed earlier, barrier‐crossing events are thermally activated and diffusion‐driven before the build‐up of viscosity of the epoxy matrix that accompanies the cross‐linking process. They are suppressed at low curing temperature (0.3 $\varepsilon k_{B}^{-1}$) (Figure 2e; Figure S3b, Supporting Information). However, during stretching which takes place at 0.3 $\varepsilon k_{B}^{-1}$ in the CGMD simulations, barrier‐crossing events do occur, implying that an alternative mechanism is required. For amorphous, highly cross‐linked epoxy network structure in response to stretching, a prevailing framework known as “shear transformation zones” (STZs) introduces discrete molecular rearrangements, in conjunction with interactions with their local surroundings, as the fundamental microstructural mechanisms driving the viscoplasticity.^[^ ^31^, ^32^, ^33^ ^]^ The random, heterogeneous activation of STZs induces energy‐barrier‐crossing processes that conform to a fractal potential energy landscape (PEL), describing the energetics and dictating configurational changes within individual STZs.^[^ ^32^, ^34^, ^35^ ^]^ We can identify regions of local molecular rearrangement by the nonaffine squared displacement ($D_{\min}^{2}$) field, where a nonzero value indicates a molecular displacement deviating from a linear elastic manner,^[^ ^33^, ^36^ ^]^ and look for inter‐nanotube junctions that bypass the polymer layer barrier under attraction via vdW interactions between neighboring nanotubes (**Figure** **4**a). In the region of relatively high nonaffine displacement (green), barrier‐crossing events that lead to an increase in current (from red to orange) due to lower inter‐nanotube separation can be correlated (Figure 4b). The occurrence of barrier‐crossing events during deformation at 0.3 $\varepsilon k_{B}^{-1}$, and their inhibition during curing at the same temperature, can be attributed to the stress‐induced flattening of a local minimum on the PEL of an STZ; this feature reduces the activation energy required for configurational changes (Figure 4d). Considering the stochastic nature of STZ activation, higher numbers of nanotube junctions results in a higher probability of local molecular rearrangement‐induced barrier‐crossing events (Figure 3e). The activation of STZs progresses with strain; thus, the resistance decreases gradually. In other words, barrier‐crossing events are locally plasticity‐enabled and stress‐driven during stretching, as opposed to the thermally activated diffusion‐driven events in a viscous medium during curing.

### 2.3 Structural‐Property Relationships

**Figure 5:**
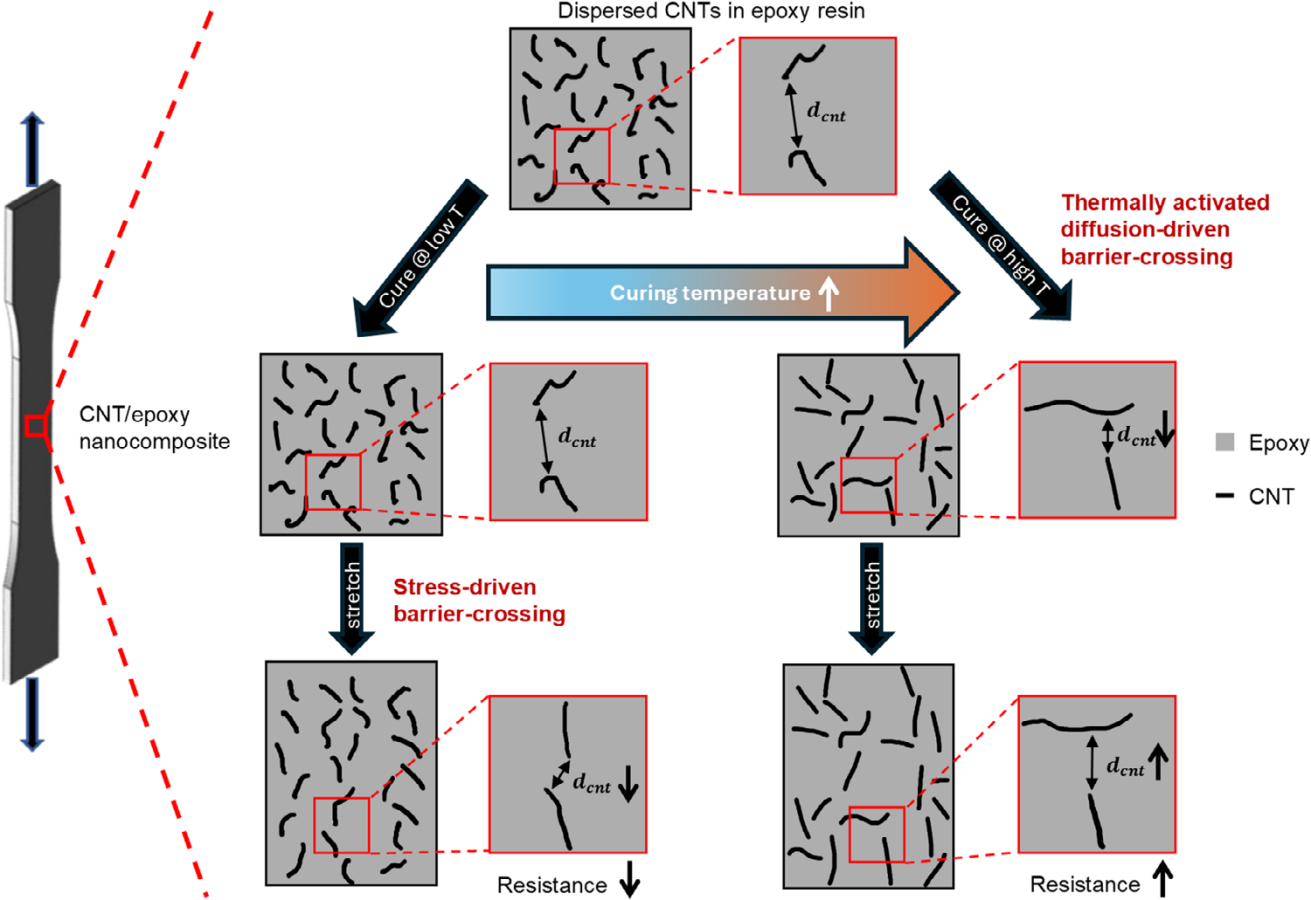
Schematic of the morphological change in CNT network induced by thermally activated diffusion‐driven and stress‐driven barrier‐crossing that determines the piezoresistive behavior of CNT/epoxy nanocomposite under tensile deformation.

Based on the agreement between the experimental results and the CGMD simulations, we identified the nanoscale dispersion state or inter‐nanofiller junction geometry as the dominant microstructural feature for the piezoresistive behavior (**Figure** **5**). Here, we further demonstrate the controllability of the monotony of the piezoresistive behavior through the inter‐nanotube junction geometry using the CGMD model. We compared the piezoresistive behaviors of the CNT/epoxy nanocomposites with different epoxy matrices, namely, DER332 and EL2, which are both bisphenol A diglycidyl ethers‐based. The curing temperatures for DER332 and EL2 matrices to attain monotonic piezoresistive behavior are 80 and 60 °C respectively (Figure 1a,b; Figure S7, Supporting Information). This suggests that the influence of the matrix on the inter‐nanofiller junction geometry is indispensable and warrants further investigation.

**Figure 6:**
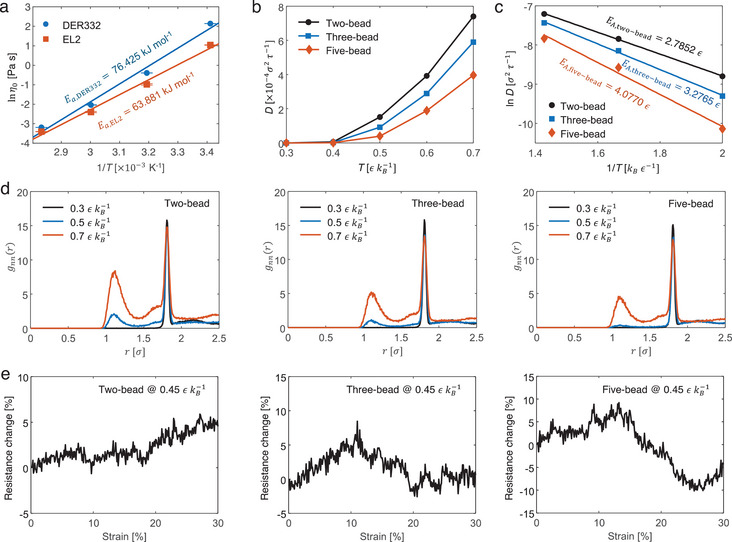
Experimental and CGMD simulation results of the influence of polymer molecular structure on inter‐nanofiller junction geometries and piezoresistive behavior in polymer nanocomposites. a) Experimental Arrhenius plots of activation energy (*E*_a_) for the viscous flow of DER332 and EL2 epoxy resins. Each data point represents the mean of three measurements (error bar: ±1 standard deviation). b) Simulated diffusivity versus temperature relationships of nanotube beads. c) Simulated Arrhenius plots of activation energy (*E*_A_) for nanotube diffusion. d) Simulated RDFs of nanotube beads at 0.3, 0.5, or 0.7 $\varepsilon k_{B}^{-1}$. e) Simulated resistance change versus strain relationships at 0.45 $\varepsilon k_{B}^{-1}$. The CGMD model setup is CNT concentration = 5 vol.%, number of beads per CNT = 100, CNT diameter = 1.0 *σ*, CNT waviness in terms of maximum deviation angle = 10°, and simulation cell size = 100 *σ*. The liquid epoxy structures are composed of two‐bead, three‐bead, and five‐bead mixtures.

In liquid epoxy resins, nanotube diffusion is dictated by the viscosity of the surrounding polymer molecules, which imposes an energy barrier.^[^ ^10^, ^37^ ^]^ Therefore, we measure the zero‐shear viscosity (*η*_₀_) of the epoxy matrices and fit the data by the Arrhenius equation to obtain the activation energy (*E*_a_) for viscous flow. Further details on zero‐shear viscosity measurements are provided in Section S4 (Supporting Information). The *η*_₀_ of DER332 and EL2 at 20 °C are 8.488 ± 0.022 and 2.824 ± 0.026 Pa⋅s respectively. This indicates that the *M*_W_ of DER332 is higher than that of EL2, as *η*_₀_ is related to molecular weight (*M*_W_), i.e., *η*_₀_ ≈*M*_W_.^[^ ^38^ ^]^ From the viscosity‐temperature relationships, the *E*_a_ for the viscous flow of DER332 (76.425 kJ mol^−1^) is higher than that of EL2 (63.881 kJ mol^−1^) (**Figure** **6**a). These results suggest that the energy activated for nanofiller diffusion would be higher in a polymer matrix with higher viscosity and molecular weight. Therefore, we vary the initial structure of the epoxy matrix in the CGMD model by replacing two‐bead resin molecules with three‐bead and five‐bead molecules (Figure S3, Supporting Information). To characterize the mobility of the nanotube under diffusion^[^ ^10^ ^]^ and describe its thermally activated nature, we fitted the diffusivity (*D*) calculated from the slopes of the mean‐square displacements using the Arrhenius equation^[^ ^32^ ^]^ to obtain the activation energy (*E*_A_) for nanotube diffusion. A detailed calculation of the activation energy is provided in Section S4 (Supporting Information). The movement of a nanotube is activated when the temperature approaches 0.5 $\varepsilon k_{B}^{-1}$ with higher mobility in mixture of shorter polymer strands (Figure 6b). The increase in diffusivity with temperature in the simulations (Figure 6b) parallels the decrease in viscosity with increasing temperature in the experiments (Figure 6a), both of which indicate enhanced molecular mobility at higher temperatures. The *E*_A_ for nanotube diffusion increases with the length of the polymer strand, from 2.7852 *ϵ* for two‐bead mixture to 3.2765 *ϵ* and 4.0770 *ϵ* for three‐bead mixture for five‐bead mixtures respectively (Figure 6c). This is analogous to the higher *E*_a_ for viscous flow in DER332 than in EL2, which requires higher curing temperatures to achieve similar CNT mobility and overcome the *E*_A_ for nanotube re‐agglomeration under diffusion. The temperature dependence of the inter‐nanotube junction geometry was affected by the molecular structure of the resin, as shown by the RDF plots for polymer strands of different lengths (Figure 6d). In particular, the height of 1σ peak decreases with longer polymer strands, indicating suppressed diffusion‐driven barrier‐crossing events.

While a direct one‐to‐one conversion between reduced units ($\varepsilon k_{B}^{-1}$) and real temperatures (°C) is not straightforward due to the coarse‐grained nature of the model,^[^ ^21^, ^23^, ^39^, ^40^ ^]^ we can provide a qualitative correlation based on the observed piezoresistive and diffusive behaviors in both simulations and experiments. The simulation temperatures of 0.3 to 0.7 $\varepsilon k_{B}^{-1}$ represent a range of thermal activation levels that influence the diffusion of CNTs. At 0.3–0.4 $\varepsilon k_{B}^{-1}$, diffusion is suppressed, leading to a well‐dispersed CNT state with polymer barriers between nanotubes, resulting in nonmonotonic piezoresistive behavior. This corresponds qualitatively to the 0.3–0.4 $\varepsilon k_{B}^{-1}$ range to the lower experimental curing temperatures (e.g., 20–60 °C). The corresponding resistance change‐strain curves at curing temperature of 0.45 $\varepsilon k_{B}^{-1}$ is monotonic in two‐bead mixture but nonmonotonic in three‐bead and five‐bead mixtures (Figure 6e), whereas the monotony remains unchanged at 0.3 and 0.7 $\varepsilon k_{B}^{-1}$ (Figure S10, Supporting Information). This corresponds to the experimental observation that EL2‐based nanocomposites exhibit monotonic piezoresistive behavior at a curing temperature of 60 °C, whereas DER332‐based nanocomposites remain nonmonotonic at the same temperature. Owing to the larger viscosity and higher *E*_a_ of DER332, a higher temperature was required to induce CNT diffusion and re‐agglomeration during the low‐viscosity stage of curing to attain monotonic piezoresistive behavior. At 0.5–0.7 $\varepsilon k_{B}^{-1}$, thermally activated diffusion enables barrier‐crossing events and CNT re‐agglomeration, leading to direct inter‐nanotube contacts and monotonic piezoresistive behavior. Specifically, the onset of significant diffusion as observed at 0.5 $\varepsilon k_{B}^{-1}$ in simulations aligns with the glass transition temperature (*T*_g_) of the CGMD model as reported in the literature,^[^ ^21^, ^22^ ^]^ which indicates a transition in material behavior analogous to real epoxy systems near typical curing temperatures. This mirrors the experimental results at higher curing temperatures (e.g., 80–100 °C).

We ran other control simulations in which nanofiller‐related parameters, including concentration, length, diameter, and waviness, were varied (Figures S11–S15, Supporting Information). The general dependence of the piezoresistive behavior on the inter‐nanofiller junction geometry remained similar. These results reinforce our proposal that the inter‐nanofiller junction geometry is the dominant factor for the monotonic piezoresistive behavior. In addition to the curing temperature and polymer molecular structure that modulate the inter‐nanofiller junction geometry through diffusion under attraction via vdW interactions, the alignment of the nanofiller in an electric field during curing^[^ ^14^ ^]^ can alternatively drive the direct contact of neighboring nanofillers owing to electrostatic interactions associated with the applied voltage.^[^ ^10^ ^]^ For nanotechnology applications, it may be possible to design and optimize strain‐sensing polymer nanocomposites based on the curing temperature dependence of the piezoresistive behavior on the viscosity of the polymer matrix. For example, a higher curing temperature may be required for epoxy resins with higher viscosity, or a less viscous resin may be adopted if a lower curing temperature is preferred.

## 3 Conclusion

This paper presents the results of an in‐depth investigation of the microstructural origin of the nonmonotonic piezoresistive behavior of polymer nanocomposites. The experimental results indicate that the piezoresistive behavior transitions from nonmonotonic to monotonic with increasing curing temperature, and the CGMD simulations reproduce this temperature dependence. Through further analysis, we successfully identified the inter‐nanofiller junction geometry as a microstructural parameter governing the monotony of the piezoresistive behavior. During curing, thermally activated diffusion at high temperatures induces re‐agglomeration of and direct contact between nanofillers in the liquid polymer matrix owing to vdW interactions. As a result, monotonic piezoresistive behavior is attained, as agglomerated nanofillers generally dissociate during stretching. For a nanofiller network developed under suppressed diffusion at low curing temperatures, re‐agglomeration of the nanofiller is stimulated during stretching by molecular rearrangements in stress‐activated STZs, which are associated with the viscoplastic deformation of the polymer matrix, leading to a decrease in resistance. The molecular structure of the polymer matrix, which affects nanofiller diffusion, also plays an important role in the modulation of the inter‐nanofiller junction geometry and piezoresistive behavior.

Modulating inter‐nanofiller junction geometry enables control over the monotony of piezoresistive behavior in polymer nanocomposites. By developing nanocomposites with monotonic piezoresistive behaviors, high‐performance strain‐sensing materials with extended operating ranges can be developed for real applications. The findings contribute to understanding electromechanical properties of polymer nanocomposites and provide a framework for designing advanced strain‐sensing materials.

## 4 Experimental Section

### Materials and Sample Fabrications

Chemical vapor deposition‐grown multiwalled carbon nanotubes (purity: > 95%, outer diameter: 50–90 nm, aspect ratio: > 100), high‐purity bisphenol A diglycidyl ether (DGEBA) liquid epoxy resin (DER332), and a polypropylene glycol‐based polyetheramine curing agent, with an average molecular weight of ≈230, were purchased from Sigma–Aldrich, USA. Another DGEBA‐based epoxy resin (EL2) and a cycloaliphatic‐amine‐based epoxy hardener (AT30 Slow) were supplied by Easy Composites (UK). The CNT/epoxy nanocomposite samples were fabricated using a mechanical approach (Figure S5a, Supporting Information). The CNT/epoxy resin mixture was mechanically stirred for 30 min at 2000 rpm using an overhead stirrer (Hei‐TORQUE Core, Heidolph Instruments, Germany) with a radial flow impeller (TR 21, Heidolph Instruments, Germany). The mixture was then sonicated in an ultrasonic bath (RS PRO Ultrasonic Cleaner; RS Components, UK) for 30 min. After blending the mixture with an epoxy hardener at a resin‐to‐hardener weight ratio of 1000:344 for the DER332/polyetheramine system and 10:3 for the EL2/AT30 Slow system, it was degassed in a vacuum oven. Finally, the CNT/epoxy resin/epoxy hardener mixture was poured into a silicone mold of dumbbell‐shaped tensile specimens and cured at various temperatures ranging from room temperature to 100 °C (Table S1, Supporting Information). The CNT content was fixed at 0.3 wt. % based on the previous work.^[^ ^19^ ^]^

### Electromechanical Tests

Quasistatic tensile tests were performed on the CNT/epoxy nanocomposite samples using a universal testing machine (5982, Instron, USA). A minimum of three specimens were tested for each sample. The specimens were loaded at a crosshead speed of 1 mm min^−1^ until failure. During loading, the electrical resistance and axial deformation of the specimens were measured simultaneously (Figure S5b, Supporting Information). Craft paper was bonded to the gripping areas of the specimens using a cyanoacrylate adhesive to electrically insulate the conductive specimens using a universal testing machine. The deformation of the specimens was calculated using machine crosshead displacement.

### Sample Characterizations

The morphology of the CNT network was imaged using an FESEM (MAIA3, TESCAN, Czech Republic) operating at 5 kV. The imaging surfaces were processed using an ultramicrotome (PowerTome‐XL, RMC Boeckler, USA) and coated with a thin layer of gold via sputtering (MCM‐200, NanoImages, USA). The electrical resistances of the CNT/epoxy nanocomposite samples were measured via a two‐probe method using a digital multimeter (DMM6500, Keithley Instruments, USA). A pair of parallel electrodes were marked using conductive silver paint (SPI Supplies, USA), 20 mm apart (Figure S5b, Supporting Information). Fourier transform infrared spectra were collected in situ using an IR spectrometer (Spectrum Two, PerkinElmer, Waltham, MA, USA) equipped with a deuterated triglycine sulfate detector. A mixture of DER332 and a polyetheramine curing agent was sandwiched between potassium bromide windows using a 2‐mm PTFE spacer. All the spectra were obtained in the region of 8000–4000 cm^−1^ with a resolution of 4 cm^−1^ and 16 scans during isothermal curing. Spectral analysis was performed using the Spectrum 10 software (PerkinElmer, USA). Dynamic rheological measurements were performed using an oscillatory rheometer (MCR702 TwinDrive Rheometer, Anton Paar, Austria) to determine the shear flow characteristics of the DER332 and EL2 epoxy matrices. A 25‐mm diameter parallel plate with a 1‐mm gap configuration was used. The steady shear viscosity was measured at a shear rate of 0.1–100 s^−1^ and temperatures ranging from 20 to 80 °C.

### Molecular Dynamic Simulations

The CGMD method was utilized to simulate and analyze the dynamic percolation of CNTs and the dynamic crosslinking process of epoxy under different temperatures, as well as the CNT movement and morphological changes of the CNT network during tensile deformation, using the MD package LAMMPS^[^ ^41^ ^]^ and a post‐processing visualization tool OVITO.^[^ ^42^ ^]^ The unit cell contained randomly dispersed CNTs and epoxy network, both of which were treated as bead‐spring chains. Each CNT was discretized into a series of beads that interacted with adjacent beads on the same CNT through a stiff finite extensible nonlinear elastic bond potential and harmonic angle potential, accounting for the stretching and bending stiffness. The epoxy matrix comprises resin and crosslinker beads, which can form bonds to develop the network structure.^[^ ^22^ ^]^ The vdW interactions between all beads were modeled using a truncated and shifted Lennard‐Jones potential. The initial configurations of the unit cell were created by randomly placing the CNTs, two‐, three‐, or five‐bead resin molecules, and crosslinker beads in a simulation box. The number ratio between the resin molecules and crosslinker beads was set using stoichiometry. All beads are allowed to overlap during generation, and the overlapping was removed by equilibration at *T* = 1.0 $\varepsilon k_{B}^{-1}$ using the Langevin thermostat and applying a cosine potential.^[^ ^22^ ^]^ Dispersion of CNTs was achieved by setting the interfacial interaction parameter between CNTs and polymer (*ε*_np_) to 2.0 *ϵ* (Figure S1, Supporting Information).^[^ ^23^ ^]^ Subsequently, to investigate the impact of curing temperature, the temperature was adjusted to 0.3, 0.5, and 0.7 $\varepsilon k_{B}^{-1}$ and equilibrate the system. This equilibration process allows the system to adapt to specified temperature and pressure conditions under an isothermal (NPT) ensemble using a Nose–Hoover temperature thermostat and pressure barostat. After equilibrium was achieved, an epoxy network was established through dynamic crosslinking at the selected temperature. Then, the system was cooled to *T* = 0.3 $\varepsilon k_{B}^{-1}$ and equilibrated in the NPT ensemble. The simulation box was deformed under uniaxial tension to investigate the response of the system to the external forces and strains. At zero strain and discrete strain increments, the position of the CNT beads was exported, and the equivalent resistance of the CNT network was calculated using a resistor network model to obtain the resistance‐strain relationship.^[^ ^18^ ^]^

### Statistical Analysis

Electromechanical tests were performed on the CNT/epoxy nanocomposite samples according to the ASTM D638 standard. Each curve in the figure represents the mean ± SD of three samples. For the dynamic rheological measurements, each data point in the figure represents the mean ± SD of the three samples. For the free‐space length analysis and electrical conductivity measurements, each data point in the figure represents the mean ± SD of the four samples.

## Conflict of Interest

The authors declare no conflict of interest.

## Supporting information



Supporting Information

## Data Availability

The data that support the findings of this study are available from the corresponding author upon reasonable request.
