# Supplementary material for: Microstructural Origin of Nonmonotonic Piezoresistivity in Polymer Nanocomposites
Source: Adv Sci (Weinh). 2025 Jun 23;12(35):e04381. doi: 10.1002/advs.202504381 (PMC12463059; doi:10.1002/advs.202504381)
Supplement: Supplementary file 1 — Supporting Information [file ADVS-12-e04381-s001.docx]

Supporting Information

**Microstructural Origin of Nonmonotonic Piezoresistivity in Polymer Nanocomposites**

*Ting Yui Wong, Kui Lin, Tao Yu*, and Fangxin Zou**

# S1. CGMD model and simulation methods

To investigate the microstructural origin of the nonmonotonic piezoresistive behavior in CNT/epoxy nanocomposites, we used the CGMD method to simulate and analyze the dynamic percolation of CNTs and the dynamic crosslinking process of epoxy at different temperatures, as well as the CNT movement and morphological changes in the CNT network under deformation. The unit cell contained randomly dispersed CNTs and an epoxy polymer matrix, both of which were represented by a bead-spring model. We adopt a reduced-unit formalism. All physical quantities are expressed as multiples of fundamental quantities, mass ($m$), energy ($\epsilon$), distance ($\sigma$) and Boltzmann constant ($k_{B}$) which are all set equal to one in the simulation. This approach has been widely adopted in molecular dynamics simulations to focus on relative interactions and dynamics rather than absolute physical values, particularly when the goal is to capture qualitative trends and mechanisms.^[1,2]^

**S1.1. Pair potential**

The nonbonded or van der Waals interactions between all beads are represented by the truncated and shifted Lennard-Jones (LJ) potential

$$\begin{aligned} U_{\text{LJ}}\left( r \right)=4\varepsilon\left[ \left( \frac{\sigma}{r-\Delta} \right)^{12}-\left( \frac{\sigma}{r-\Delta} \right)^{6} \right]\#\left( \text{S}\text{}\text{ SEQ Equation \textbackslash* ARABIC }\text{}\text{1}\text{} \right) \end{aligned}$$

where $r$ is the distance between beads, $\varepsilon$ is the interaction parameter or the depth of the potential, $\sigma$ is the distance parameter or the zero-crossing distance for the potential, and $\Delta$ is the shift factor for the change in the size of bead, with a cutoff distance of $2.5 \sigma$. Each CNT or polymer chain is discretized into a series of beads that interact with adjacent bonded beads on the same molecule, the stretching stiffness of which is accounted for by a stiff finite extensible nonlinear elastic (FENE) bond potential.

$$\begin{aligned} U_{\text{FENE}}(r)=-0.5K_{\text{FENE}}R_{0}^{2}\ln\left[ 1-\left( \frac{r-\Delta}{R_{0}} \right)^{2} \right]\#\left( \text{S}\text{}\text{ SEQ Equation \textbackslash* ARABIC }\text{}\text{2}\text{} \right) \end{aligned}$$

where $K_{\text{FENE}}$ is the coefficient for the stretching stiffness of the bond, and $R_{0}=1.5 \sigma$ is the equilibrium bond length. The stretching stiffness coefficients for the polymer and CNT chains are set as $30 \epsilon\sigma^{-2}$ and $80 \epsilon\sigma^{-2}$ respectively to reflect the higher stretching stiffness of CNT than polymer. The bending stiffness of the CNT was introduced using a harmonic angle potential:

$\begin{aligned} U_{\text{angle}}\left( \theta\right)=K_{\text{angle}}\left( \theta-\theta_{0} \right)^{2}\#\left( \text{S}\text{}\text{ SEQ Equation \textbackslash* ARABIC }\text{}\text{3}\text{} \right) \end{aligned}$

where $\theta$ is the bond angle of CNT beads, $K_{\text{angle}}=10 \epsilon\sigma^{-2}$ is the coefficient for the bending stiffness of the chain, and $\theta_{0}={180}^{\text{o}}$ is the equilibrium value of the angle potential. These parameters (e.g., interaction strength *ϵ*, bond stiffness *K*_FENE_, and bending stiffness *K*_angle_) are tuned to qualitatively represent the relative differences in interactions (e.g., higher stiffness for CNTs compared to polymer chains) rather than to match exact physical ratios. In addition, the mass of a CNT bead (*m*_CNT_) was set to 2 *m* when the diameter was 1 *σ* and scaled with its diameter to better reflect the differences in the CNT and polymer matrices.

**S1.2 Simulation methods**

The initial configurations of the CNTs are randomly created with the relative orientation of consecutive bonds ($\theta-\theta_{0}$) less than a maximum deviation angle to account for the waviness of CNT.^[1]^ The simulation boxes are then filled by a liquid mixture of two-bead, three-bead or five-bead chains representing the epoxy resin molecules and crosslinker beads to a desired number density of $\sim1 \sigma^{-3}$.^[2]^ The functionalities of the epoxy and crosslinker beads were set to 1 and 4, respectively, to simulate a typical DGEBA/diamine epoxy system. The number ratio between the resin molecules and crosslinker beads was set using stoichiometry.

The overlap between the randomly placed beads was first removed by applying a cosine-pairwise potential

$\begin{aligned} U_{\text{soft}}=A\left[ 1+\cos\left( \frac{\pi r}{r_{\text{cutoff}\text{,soft}}} \right) \right]\#\left( \text{S}\text{}\text{ SEQ Equation \textbackslash* ARABIC }\text{}\text{4}\text{} \right) \end{aligned}$

where $r_{\text{cutoff}\text{,soft}}$ is the cutoff distance, and is set as the bead diameter. The amplitude $A$ is increased from 0.0 to 30.0 $\epsilon\sigma^{-2}$ over the span of 500,000 time steps for the overlapping removal stage. Then, the nonbonded interaction is switched back to the LJ potential and equilibrated at $T=1.0 \epsilon k_{B}^{-1}$ for 500,000 time steps. To disperse the CNTs, we set the interaction parameter between CNT and polymer beads $\varepsilon_{np}$ to 2.0 $\epsilon$ ^[3]^ and further run the simulations for 500,000 time steps. The dispersed state is verified by a single strong peak at approximately $r=\sqrt{3} \sigma$ in the radial distribution function of the CNT beads $g_{nn}\left( r \right)$, as shown in Figure S1. Subsequently, the systems are cooled to the curing temperature, which is varied from 0.3 to 0.7 $\epsilon k_{B}^{-1}$, over 250,000 time steps. After setting $\varepsilon_{np}$ to 1.0 $\epsilon$, further equilibrium is performed at their respective curing temperatures for 1,000,000 time steps.

**Figure S1.** Radial distribution function of CNT beads $g_{nn}(r)$ for interaction parameter between CNT and polymer beads $\varepsilon_{np}$ set to 2.0 $\epsilon$.

Liquid mixtures of resin molecules and crosslinker beads were dynamically crosslinked to form a three-dimensional molecular network of the epoxy.^[4]^ Bonds between the epoxy beads and crosslinker beads are formed when their separation is less than 1.3 $\sigma$. Possible bonding sites are checked every 10 time steps, and a probability of 0.1 for bond formation is assigned to avoid drastic changes in the system energy. This dynamic crosslinking process was conducted until a conversion degree of 95% was reached, or stopped when no more bonds were formed within 100 time steps. Thereafter, the systems are quenched from their respective curing temperature to 0.3 $\epsilon k_{B}^{-1}$, which is below the glass transition temperature of such CGMD model for epoxy (0.5 $\epsilon k_{B}^{-1}$)^[2]^, over a span of 500,000 time steps and equilibrated for 3,000,000 time steps.

To simulate the piezoresistive behavior, tensile deformation is applied by elongating the simulation cell along the $x$ direction at a strain rate of $2\times{10}^{-5} \tau^{-1}$, where $\tau=\sqrt{m\sigma^{2}/\epsilon}$ is the reduced unit of time^[5]^, while adjusting the cell sizes in the $y$ and $z$ directions to maintain zero-stress state. Periodic boundary conditions were applied in all the three directions throughout the simulation. The configurations of the CNT networks are outputted every 10,000 time steps or $50 \tau$ for calculation of electrical resistance using a resistor network model, as discussed in Section S2.

# S2. Resistor network model for calculating the resistance of CNT network in CGMD simulation

The resistance-strain relationship was obtained by calculating the electrical resistance of the CNT network in discrete strain increments using the equivalent resistor network method.^[6]^ The CNT network was transformed into an equivalent resistor circuit with each CNT bead as a node in the circuit such that nodal analysis could be applied to calculate the electrical resistance (Figure S2). Two transport mechanisms, intrinsic conductance and the tunnelling effect, are considered to contribute to the electrical conduction path of the CNT network. In the intrinsic conductance mechanism, charge transport along each CNT is described by a series of resistors connecting neighboring nodes with resistance, given by

$\begin{aligned} R_{\text{node}}=\frac{R_{\text{CNT}}}{N_{\text{node}}-1}\#\left( \text{S} SEQ Equation \backslash* ARABIC 5 \right) \end{aligned}$

where $R_{\text{CNT}}$ is the total resistance of a CNT and $N_{\text{node}}$ is the number of nodes per CNT. Notably, in a given configuration, all the CNTs have the same length or number of nodes. We assign $R_{\mathrm{CNT}}=17.3 k\Omega$ even though the overall resistance change is largely unaffected by $R_{\text{node}}$ or $R_{\text{CNT}}$, which, in reality, is multiple orders of magnitude smaller than the tunnelling resistance $R_{\text{tunnel}}$.^[6]^ For the tunnelling mechanism, when the distance between the CNT beads from two CNTs is smaller than the tunnelling cutoff distance $d_{\text{cutoff}}$, which is set as $2.5 \sigma$, the charge transport between the CNT pair is described by a resistor connecting the node pair with the lowest separation $d_{\text{tunnel}}$, given by

$$\begin{aligned} R_{\text{tunnel}}=\frac{h}{2e^{2}}\frac{1}{MT_{\text{prob}}}\#\left( \text{S} SEQ Equation \backslash* ARABIC 6 \right) \end{aligned}$$

where $h$ is Planck’s constant, $e$ is the electron charge, and $M=400$ is the total number of conduction channels.^[7]^ The transmission probability $T_{\text{prob}}$ was estimated by solving the Schrödinger equation with a rectangular potential barrier as follows:

$\begin{aligned} T_{\text{prob}}=\text{exp}\left( -\frac{d_{\text{tunnel}}}{d_{\text{char}}} \right)\#\left( \text{S}\text{}\text{ SEQ Equation \textbackslash* ARABIC }\text{}\text{7}\text{} \right) \end{aligned}$

with the tunnelling characteristic length given by

$\begin{aligned} d_{\text{char}}=\frac{h}{2\pi\sqrt{8m_{\text{e}}W}}\#\left( \text{S}\text{}\text{ SEQ Equation \textbackslash* ARABIC }\text{}\text{8}\text{} \right) \end{aligned}$

where $m_{\text{e}}$ is the electron mass and $W=1.5 \text{eV}$ is the work function difference between the CNTs and the polymer barrier.^[8]^ The tunnelling distance $d_{\text{tunnel}}$ was transformed from LJ units to real (SI) units as follows:

$\begin{aligned} d_{\text{tunnel,real}}=d_{\text{vdW,real}}+\frac{\left( d_{\text{cutoff,real}}-d_{\text{vdW,real}} \right)\times\left( d_{\text{tunnel,LJ}}-d_{\text{vdW,LJ}} \right)}{d_{\text{cutoff,LJ}}-d_{\text{vdW,LJ}}}\#\left( \text{S} SEQ Equation \backslash* ARABIC 9 \right) \end{aligned}$

where $d_{\text{vdW,real}}=0.34 \text{nm}$ is the van der Waals distance between CNTs in real unit, $d_{\text{cutoff,real}}=1.0 \text{nm}$ is the tunnelling cutoff distance in real unit^[8]^, and $d_{\text{vdW,LJ}}=2^{1/6} \sigma$ is the van der Waals distance between CNTs in LJ unit. Periodic boundary conditions were applied in the $y$ and $z$ directions to minimize the influence of the simulation cell size.^[6,9]^

**Figure S2.** Schematic of the equivalent resistor network model.

According to Kirchhoff’s current law, the elemental matrix representation of the relation between the external input current $I$ passing through a resistor connecting nodes $i$ and $j$ and the nodal voltage $V$ is

$\begin{aligned} \left\{ {I_{i}^{e} \atop I_{j}^{e}} \right\}=\left[ K_{ij}^{e} \right]\left\{ {V_{i} \atop V_{j}} \right\}=\frac{1}{R_{ij}^{e}}\left[ \begin{matrix} 1 & -1 \\ -1 & 1 \end{matrix} \right]\left\{ {V_{i} \atop V_{j}} \right\}\#\left( \text{S} SEQ Equation \backslash* ARABIC 10 \right) \end{aligned}$

where $K_{ij}^{e}$ is the element conductance and $R^{e}$ is the element resistance.^[10]^ The elemental matrices for all the resistors were then assembled into a system of algebraic equations for the entire CNT network.

$$\begin{aligned} \text{I}\text{=}\text{KV }\boldsymbol{\#}\text{(}\text{S}\text{}\text{ SEQ Equation \textbackslash* ARABIC }\text{}\text{11}\text{}\text{)} \end{aligned}$$

where $\text{V}$ $=\left\{ V_{1}, V_{2},\ldots, V_{n} \right\}^{T}$ is the vector of the nodal voltages, $\text{I}$ $=\left\{ I_{1}, I_{2},\ldots, I_{n} \right\}^{T}$ is the vector of the external input current, and is the global conductance matrix.

$$\begin{aligned} \text{K}=\sum_{e=1}^{m} \left[ K_{ij}^{e} \right]\#\left( S SEQ Equation \backslash* ARABIC 12 \right) \end{aligned}$$

where $m$ is the number of resistance elements. To solve Equation $\text{(}\text{S11})$, we assumed that a source voltage $V_{\mathrm{source}}$ of $1 \text{V}$ was applied at the two boundaries of the loading direction; that is, at the two $y$-$z$ surfaces. Under the periodic boundary condition and applied voltage, an external input current term is introduced at any node $i$ near the high-potential boundary (i.e., the left $y$-$z$ surface) that is bonded to a node near the low-potential boundary (i.e., the right $y$-$z$ surface) as $I_{i}=I_{\mathrm{source}}=V_{\mathrm{source}}/R_{\mathrm{node}}$ (e.g., for the model shown in Figure S2, an external input current term is introduced at Node 1, which is bonded to Node 10, and Node 11, which is bonded to Node 13). After solving for the nodal voltages, the total current passing through the simulation cell is calculated using the total current of the nodes connected across the high-potential boundary (e.g., nodes 1 and 11) as^[6]^

$$\begin{aligned} I_{\text{total}}=\sum_{e=1}^{m} \frac{1}{R_{ij}^{e}}\left( V_{\mathrm{source}}-V_{i} \right)\#\left( \text{S}\text{}\text{ SEQ Equation \textbackslash* ARABIC }\text{}\text{13}\text{} \right) \end{aligned}$$

Finally, the equivalent resistance of the CNT network is obtained using Ohm’s law.

$$\begin{aligned} R_{\text{equivalent}}=\frac{V_{\mathrm{source}}}{I_{\text{total}}}\#\left( \text{S}\text{}\text{ SEQ Equation \textbackslash* ARABIC }\text{}\text{14}\text{} \right) \end{aligned}$$

# S3. Quantitative characterization of dispersion state of CNTs using free-space length analysis and electrical conductivity measurement

To capture the influence of barrier crossing events on the inter-nanotube junction geometry, we used SEM with free-space length analysis^[11]^ and electrical conductivity measurements^[12]^ to quantitatively characterize the dispersion state of the CNTs. Khare and Burris adopted a method for quantifying the dispersion state.^[11]^ Its principle is to compute the free-space length $L_{f}$ which correlates with the continuous area free of nanoparticles. Specifically, statistically substantial numbers of squares of different sizes were randomly positioned throughout the SEM image to create a histogram displaying the probability that a given number of nanoparticles existed within a square (e.g., in this work, 10 000 squares of each square size were used). The free-space length $L_{f}$ is defined as “the largest square size for which the most probable number of intersecting nanoparticles in a randomly placed square is zero.” The electrical conductivity of each sample was calculated as follows:

$$\begin{aligned} \rho=\frac{D}{A\cdot R}\#\left( \text{S}\text{}\text{ SEQ Equation \textbackslash* ARABIC }\text{}\text{15}\text{} \right) \end{aligned}$$

where $D$ is the distance between the two electrodes, $A$ is the cross-sectional area of the sample, and $R$ is the measured resistance of the sample.

For uniform dispersion, nanoparticles are distributed in a regular fashion throughout the micrograph, so the space between nanoparticles or agglomerates, and hence the free-space length, is minimized. In a system near the percolation threshold like our CNT/epoxy nanocomposite, uniform dispersion leads to the presence of an insulating polymer layer surrounding each nanoparticle and lower electrical conductivity, as in the case of the 60 and 70 °C cured samples (Figure 2c). However, when nanoparticles migrate closer to each other in local agglomerates, continuous areas free of nanoparticles emerge and expand between the agglomerates, leading to a larger free-space length. Local agglomerates of nanoparticles facilitate the formation of electrical conduction paths, resulting in higher electrical conductivity. It can be observed from Figure 2c that an increase in curing temperature leads to larger free-space lengths and higher conductivities in the 80 and 100 °C samples compared to the 60 and 70 °C samples.

The above procedures are repeated on the CGMD simulations, using the images of slices with thickness of $1 \sigma$ to mimic the FESEM images (Figure 2b). The electrical conductivity was calculated as discussed in Section S2. An increase in the curing temperature led to a higher conductivity and free-space length, resembling the experimental data (Figure 2d).

# S4. Influence of epoxy resin structure on thermally activated diffusion of nanotubes, and temperature dependence of inter-nanotube junction geometry and piezoresistive behavior

Dynamic rheological measurements were performed to determine the shear flow characteristics of epoxy matrices DER332 and EL. A 25-mm diameter parallel-plate and 1-mm gap configuration were used. The steady shear viscosity was measured at a shear rate in the range of 0.1–100 s^−1^ and at temperatures ranging from 20 to 80 °C. Both the DER332 and EL2 epoxy resins exhibited Newtonian behavior, that is, shear-rate-independent viscosity. The thermal activation energy $E_{a}$ for a viscous flow can be calculated using the Arrhenius model:

$$\begin{aligned} \ln\eta_{0}= \ln A+\frac{E_{a}}{RT}\#\left( S SEQ Equation \backslash* ARABIC 16 \right) \end{aligned}$$

where $\eta_{0}$ is the zero-shear viscosity, $A$ is the prefactor, $R$ is the gas constant, and $T$ is the temperature. Owing to the Newtonian behavior, $\eta_{0}$ can be taken as the viscosity measured. The $E_{a}$ for the viscous flow of DER332 (76.425 kJ mol^−1^) was higher than that of EL2 (63.881 kJ mol^−1^), as shown in Figure 5a. These values are consistent with those reported previously.^[13,14]^

We calculated the activation energy $E_{\text{A}}$ for the diffusion of CNTs in liquid two-bead, three-bead, and five-bead epoxy mixtures^[15,16]^ (Figure S3a). From the CGMD simulations, diffusivity *D* is related to the mean square displacement (MSD) by

$$\begin{aligned} D= \lim_{t\to\infty} \frac{1}{6t}\left\langle\left| \vec{r}\left( t \right)-\vec{r}\left( 0 \right) \right|^{2} \right\rangle\#\left( S SEQ Equation \backslash* ARABIC 17 \right) \end{aligned}$$

where $\vec{r}\left( 0 \right)$ and $\vec{r}\left( t \right)$ represent the position vectors of an atom at the initial time and time *t* respectively, and < > is the average over all nanotube beads. The value of *D* was obtained from the slope of the MSD vs. time curve, as shown in Figure S3b. The diffusivities for the nanotube beads at *T* = 0.3, 0,4, 0.5, 0.6, and 0.7 $\epsilon k_{B}^{-1}$ are calculated and compared in Figure 5b. The diffusion is thermally activated at *T* = 0.5 $\epsilon k_{B}^{-1}$ regardless of the epoxy resin structure, whereas it becomes increasingly restricted in three-bead and five-bead mixtures.

We assume that the temperature dependence of the diffusivity of nanotubes can be described by the Arrhenius form of a thermally activated process:

$$\begin{aligned} \ln D= \ln D_{0}-\frac{E_{A}}{k_{B}T}\#\left( S SEQ Equation \backslash* ARABIC 18 \right) \end{aligned}$$

where $D_{0}$ is the prefactor. The energy barrier $E_{A}$ can be obtained from the slope of $\ln D$ vs. $1/T$ for the range of 0.5 to 0.7 $\epsilon k_{B}^{-1}$, in which barrier-crossing events are activated. The results are shown in Figure 6c, which indicates that the $E_{A}$ for the diffusion of nanotube beads increases with the length of the polymer strand.

**Figure S3.** Influence of epoxy molecular structure on mobility and thermally activated diffusion of CNTs. a) Schematics of two-bead, three-bead, and five-bead polymer strands. b) MSD against time step for the nanotube beads at 0.3, 0.5, and 0.7 $\epsilon k_{B}^{-1}$ in two-bead, three-bead, and five-bead mixture.

# S5. Influence of crosslinking dynamics on dynamic percolation by diffusion of nanotubes and inter-nanotube junction geometry

The observation of a lower free-space length and electrical conductivity in the 100 °C sample compared to the 80 °C sample seems to counter-argue our proposed microstructural origin for the nonmonotonic piezoresistive behavior of CNT/epoxy nanocomposites. It is important to explain this observation in order to validate our claim. A possible explanation is related to the crosslinking dynamics of the epoxy as the network development process determines the physical environment experienced by the nanotubes undergoing diffusion.

Fourier-transform infrared (FTIR) spectroscopy was used to monitor monomer conversion during the curing process of the epoxy (Methods) and gain insight into the crosslinking dynamics of the matrix DER332/polyetheramine system.^[17,18]^ Upon mixing the epoxy resin and diamine hardener in a molar ratio of 2:1, the reaction between the epoxy group and the primary amino group forms a secondary amino group (linear segment). Further reaction between another epoxy group and a secondary amino group produces a tertiary amino group (crosslinking). The concentrations of epoxy groups ([E]), primary ([A_1_]), secondary ([A_2_]), and tertiary amino groups ([A_3_]) can be estimated based on the absorbance changes at approximately 4532 and 4940 cm^−1^, which correspond to the epoxy and primary amino groups, respectively, according to the Lambert-Beer law. As shown in Figure S4a, both the epoxy group and primary amino group bands clearly decreased with curing time. The concentrations of all the monomers at any instant can be determined by

$$\begin{aligned} \left[ \text{A}_{2} \right]=\left[ E \right]_{0}\left( \beta B-\alpha\right)\#\left( S SEQ Equation \backslash* ARABIC 19 \right) \end{aligned}$$

$\begin{aligned} \left[ A_{3} \right]=\left[ E \right]_{0}\left( \alpha-\beta\frac{B}{2} \right) \#(\text{S} SEQ Equation \backslash* ARABIC 20)\#\#\# \end{aligned}$

where $\alpha=\frac{\left[ E \right]_{0}-\left[ \text{E} \right]}{\left[ E \right]_{0}}$ is the degree of epoxy conversion, $\beta=\frac{\left[ \text{A}_{1} \right]_{0}-\left[ \text{A}_{1} \right]}{\left[ \text{A}_{1} \right]_{0}}$ is the degree of primary amine conversion, and $B=\frac{2\left[ A_{1} \right]_{0}}{\left[ \text{E} \right]_{0}}$ is the ratio between the initial concentration of primary amino group and epoxy group. The initial concentration of the epoxy group $\left[ \text{E} \right]_{0}$ can be calculated from the epoxy equivalent weight (EEW),^[19]^ and that of the primary amino group $\left[ \text{A}_{1} \right]_{0}$ can be determined from the molar feed ratio between the epoxy and amine hardener. The values of $\left[ \text{A}_{2} \right]$ and $\left[ \text{A}_{3} \right]$ were obtained from the experimentally available values of $\left[ \text{E} \right]$ and $\left[ \text{A}_{1} \right]$ from their respective absorbance bands at 4532 and 4940 cm^−1^ using Equation (S19) and (S20).

Figure S4b–d show the epoxy conversion and concentrations of $\left[ \text{A}_{1} \right]$, $\left[ \text{A}_{2} \right]$, and $\left[ \text{A}_{3} \right]$ as functions of curing time for the DER332/polyetheramine/CNT system cured at 80 and 100 °C. The reaction rate is apparently higher at 100 °C than at 80 °C as indicated by the more rapid increase in epoxy conversion and the shorter time to reach plateau. Specifically, the resulting tertiary amino groups (A_3_) or crosslinks, which represent the growth of the network structure, increase viscosity and suppress diffusion.^[17]^ It can be observed that the production of crosslink or the development of network structure is more rapid at 100 °C. The $\left[ \text{A}_{3} \right]$ values level off at 180 and 60 min at 80 and 100 °C respectively. The level-off time of $\left[ \text{A}_{3} \right]$ is related to the gelation time, which indicates that an infinite molecular network of epoxy is formed, and diffusion ceases.^[18]^

**Figure S4.** FTIR results of the curing process of DER332/polyetheramine/CNT mixture. a) Time evolution of FTIR spectra for the curing process of DER332/polyetheramine/CNT mixture. b) Epoxy conversion against curing time for the DER332/polyetheramine/CNT mixture at 80 and 100 °C. c,d) Concentration of primary, secondary, and tertiary amino groups against curing time at 80 and 100 °C.

Epoxy curing is a chemical crosslinking process that leads to a continuous increase in viscosity. The nanotubes are mobile and form more connected networks in the low-viscosity stage of the curing cycle before crosslinking halts the movement of nanotubes, and thus, the dynamic percolation process.^[20,21]^ Although a higher temperature can enhance molecular mobility and promote the formation of a percolation network, it simultaneously increases the rate of crosslinking and prematurely suppresses dynamic percolation. Therefore, the lower free-space length and electrical conductivity may be attributed to the shorter time window for the barrier-crossing events that drive the changes in inter-nanotube junction geometry at 100 °C. This may also account for the lower sensitivity of the 100 °C sample.


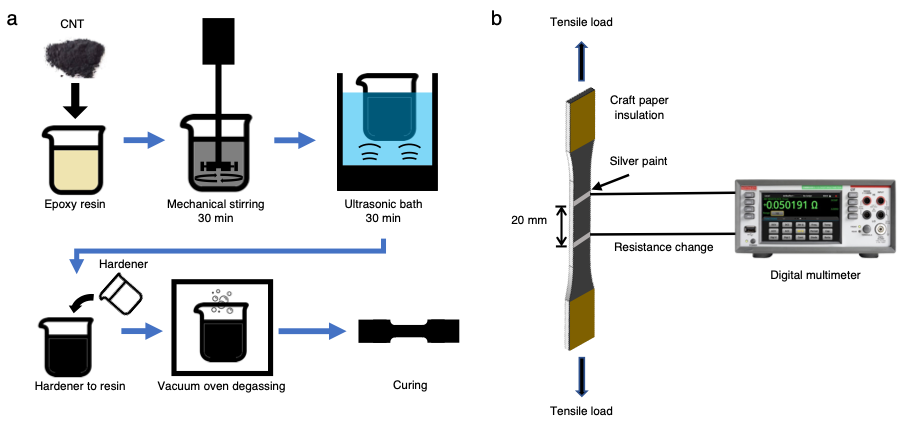


**Figure S5.** Fabrication and electromechanical test setup. a) Fabrication procedures of CNT/epoxy nanocomposite samples. b) Experimental setup for quasi-static tensile tests with simultaneous electrical resistance measurements.

**Figure S6.** Experimental results of stress versus strain relationships of CNT/epoxy nanocomposites. a-d) Experimental stress versus strain relationships of CNT/epoxy nanocomposites cured at 60 °C for 6 h (a), 70 °C for 4 h (b), 80 °C for 3 h (c) or 100 °C for 1 h (d), all post-cured at 125 °C for 3 h. For each sample, three specimens were tested, and the mean response is plotted (shaded area: ±1 standard deviation).

**Figure S7.** Experimental results of piezoresistive behavior of CNT/EL2 nanocomposite. a,b) Experimental resistance change versus strain relationships of CNT/EL2 nanocomposites cured at 25 °C for 24 h and post-cured at 60 °C for 6 h (a), cured at 60 °C for 6 h, or cured at 80 °C for 6 h (b). At least three specimens were tested for each sample and the mean response is plotted (shaded area: ±1 standard deviation).

**Figure S8.** Experimental electrical resistance of CNT/EL2 nanocomposites at different CNT concentrations. At least three specimens were tested for each sample (error bar: ±1 standard deviation).

**Figure S9.** Variation in experimental resistance with strain of CNT/EL2 nanocomposites with CNT contents of a,d) 0.1 wt.%, b,e) 0.2 wt.%, and c,f) 0.3 wt.%, cured at a–c) 25 °C for 24 h, followed by post-curing at 60 °C for 6 h, and d–f) 60 °C for 6 h. At least three specimens were tested for each sample and the mean response is plotted (shaded area: ±1 standard deviation).

**Figure S10.** CGMD simulation results of the effect of liquid epoxy structure on piezoresistive behavior of CNT/epoxy nanocomposites. a–d) Simulated resistance change versus strain relationships of CNT/epoxy nanocomposites with three-bead mixture (a,d) or five-bead mixture (c,d).

**Figure S11.** CGMD simulation results of the effect of simulation cell size on curing temperature-dependent piezoresistive behavior of CNT/epoxy nanocomposite. a–d) Simulated resistance change versus strain relationships of CNT/epoxy nanocomposites obtained with cell size of 50 *σ* (a,b) or cell size of 100 *σ* (c,d).

**Figure S12.** CGMD simulation results of the effect of CNT concentration on curing temperature-dependent piezoresistive behavior of CNT/epoxy nanocomposite. a,b) Simulated resistance change versus strain relationships of CNT/epoxy nanocomposites with CNT concentration of 4 vol.% (a,b) or CNT concentration of 6 vol.% (c,d).

**Figure S13.** CGMD simulation results of the effect of CNT length on curing temperature-dependent piezoresistive behavior of CNT/epoxy nanocomposite. a–d) Simulated resistance change versus strain relationships of CNT/epoxy nanocomposites with CNT length of 50 beads per chain (a,b) or CNT length of 200 beads per chain (c,d).

**Figure S14.** CGMD simulation results of the effect of CNT diameter on curing temperature-dependent piezoresistive behavior of CNT/epoxy nanocomposite. a–d) Simulated resistance change versus strain relationships of CNT/epoxy nanocomposites with CNT diameter of 1.5 σ (a,b) or CNT diameter of 2 σ (c,d).

**Figure S15.** CGMD simulation results of effect of the CNT waviness on curing temperature-dependent piezoresistive behavior of CNT/epoxy nanocomposites. a–d) Simulated resistance change versus strain relationships of CNT/epoxy nanocomposites with CNT waviness of 30° (a,b) or CNT waviness of 60° (c,d).

**Table S1.** Processing conditions of CNT/epoxy nanocomposite samples.

| Samples^a)^ | Curing temperature [°C] | Curing time [h] | Post-curing temperature [°C] | Post-curing time [h] |
| --- | --- | --- | --- | --- |
| CNT/DER332 | 60 | 6 | 125 | 3 |
|  | 70 | 4 |  |  |
|  | 80 | 3 |  |  |
|  | 100 | 1 |  |  |
| CNT/EL2 | 25 | 24 | 60 | 6 |
|  | 60 | 6 | n/a | |
|  | 80 | 6 |  |  |

^a)^The filler concentration is 0.3 wt.% for all samples.

# References

[1] M. A. S. Matos, V. L. Tagarielli, P. M. Baiz-Villafranca, S. T. Pinho, *J Mech Phys Solids* **2018**, *114*, 84.

[2] M. Tsige, C. D. Lorenz, M. J. Stevens, *Macromolecules* **2004**, *37*, 8466.

[3] Y. Gao, D. Cao, J. Liu, J. Shen, Y. Wu, L. Zhang, *Physical Chemistry Chemical Physics* **2015**, *17*, 22959.

[4] S. Yang, J. Qu, *Phys Rev E Stat Nonlin Soft Matter Phys* **2014**, *90*, 1.

[5] M. Panico, S. Narayanan, L. C. Brinson, *Model Simul Mat Sci Eng* **2010**, *18*.

[6] L. Jin, A. Chortos, F. Lian, E. Pop, C. Linder, Z. Bao, W. Cai, *Proc Natl Acad Sci U S A* **2018**, *115*, 1986.

[7] M. B̈üttiker, Y. Imry, R. Landauer, S. Pinhas, *Phys Rev B* **1985**, *31*, 6207.

[8] N. Hu, Y. Karube, C. Yan, Z. Masuda, H. Fukunaga, *Acta Mater* **2008**, *56*, 2929.

[9] W. S. Bao, S. A. Meguid, Z. H. Zhu, M. J. Meguid, *Nanotechnology* **2011**, *22*.

[10] C. Li, T. W. Chou, *J Phys A Math Theor* **2007**, *40*, 14679.

[11] H. S. Khare, D. L. Burris, *Polymer (Guildf)* **2010**, *51*, 719.

[12] G. Faiella, V. Antonucci, S. T. Buschhorn, L. A. S. A. Prado, K. Schulte, M. Giordano, *Compos Part A Appl Sci Manuf* **2012**, *43*, 1441.

[13] I. El Sawi, P. A. Olivier, P. Demont, H. Bougherara, *J Appl Polym Sci* **2012**, *126*, 358.

[14] D. Zhang, C. Saukas, Y. He, R. Wang, A. I. Taub, *J Mater Sci* **2020**, *55*, 16220.

[15] M. Maździarz, J. Rojek, S. Nosewicz, *Philosophical Magazine* **2018**, *98*, 2257.

[16] X. W. Zhou, R. E. Jones, J. Gruber, *Comput Mater Sci* **2017**, *128*, 331.

[17] C. M. Sahagun, S. E. Morgan, *ACS Appl Mater Interfaces* **2012**, *4*, 564.

[18] M. Aoki, A. Shundo, R. Kuwahara, S. Yamamoto, K. Tanaka, *Macromolecules* **2019**, *52*, 2075.

[19] M. Pramanik, S. K. Mendon, J. W. Rawlins, *Polym Test* **2012**, *31*, 716.

[20] C. S. Boland, U. Khan, G. Ryan, S. Barwich, R. Charifou, A. Harvey, C. Backes, Z. Li, M. S. Ferreira, M. E. Möbius, R. J. Young, J. N. Coleman, *Science (1979)* **2016**, *354*, 1257.

[21] X. Zhang, H. Sun, C. Yang, K. Zhang, M. M. F. Yuen, S. Yang, *RSC Adv* **2013**, *3*, 1916.
